# Supplementary material for: The RNA secondary structure of androgen receptor-FL and V7 transcripts reveals novel regulatory regions
Source: Nucleic Acids Res. 2024 Mar 30;52(11):6596–613. doi: 10.1093/nar/gkae220 (PMC11194067; doi:10.1093/nar/gkae220)
Supplement: gkae220_Supplemental_Files [file gkae220_supplemental_files.zip › Supplementary File S12.zip!/Supplementary File S12/Mass_Spec_analysis_settings.docx]

====================================================================================================

Samples & Files

====================================================================================================

================================================================================

Factors

================================================================================

None

================================================================================

Quan Methods

================================================================================

None

================================================================================

Files

================================================================================

[F1] P1290-01_M4_WT

D:\P1266_QE_FY2024\P1290\P1290-01_M4_WT.raw

================================================================================

Samples

================================================================================

[S1] P1290-01_M4_WT

================================================================================

Files to Samples

================================================================================

[F1] P1290-01_M4_WT

[S1] P1290-01_M4_WT

====================================================================================================

Analysis Settings

====================================================================================================

================================================================================

Consensus Step : Workflow

================================================================================

Result name: P1290-01_M4_WT

Result file: D:\P1266_QE_FY2024\P1290\P1290-01_M4_WT-(2).pdResult

Description: Result filtered for high confident peptides, with enhanced peptide and protein annotations. Add FASTA file with common contaminants to the Protein Marker node.

Workflow based on template: CWF_ProteinID_Comprehensive_Enhanced Annotation

Creation date: 9/7/2023 7:18:01 AM

Created with Discoverer version: 3.0.0.757

------------------------------------------------------------------

The workflow tree:

------------------------------------------------------------------

|-(0) MSF Files

|-(1) PSM Grouper

|-(2) Peptide Validator

|-(3) Peptide and Protein Filter

|-(4) Protein Scorer

|-(7) Protein FDR Validator

|-(5) Protein Grouping

|-(6) Peptide in Protein Annotation

|-(9) Protein Marker

|-(8) Protein Annotation

Post-processing nodes:

--------------------------------

|-(10) Result Statistics

|-(11) Display Settings

|-(12) Data Distributions

------------------------------------------------------------------

Processing node 0: MSF Files

------------------------------------------------------------------

1. Storage Settings:

- Spectra to Store: Identified or Quantified

- Feature Traces to Store: All

2. Merging of Identified Peptide and Proteins:

- Merge Mode: Globally by Search Engine Type

3. FASTA Title Line Display:

- Reported FASTA Title Lines: Best match

- Title Line Rule: standard

4. PSM Filters:

- Maximum Delta Cn: 0.05

- Maximum Rank: 0

- Maximum Delta Mass: 0 ppm

Hidden Parameters:

- MSF File(s): D:\P1266_QE_FY2024\P1290\P1290-01_M4_WT-(2).msf

------------------------------------------------------------------

Processing node 1: PSM Grouper

------------------------------------------------------------------

1. Peptide Group Modifications:

- Site Probability Threshold: 75

------------------------------------------------------------------

Processing node 2: Peptide Validator

------------------------------------------------------------------

1. General Validation Settings:

- Validation Mode: Automatic (Control peptide level error rate if possible)

- Target FDR (Strict) for PSMs: 0.01

- Target FDR (Relaxed) for PSMs: 0.05

- Target FDR (Strict) for Peptides: 0.01

- Target FDR (Relaxed) for Peptides: 0.05

2. Specific Validation Settings:

- Validation Based on: q-Value

- Target/Decoy Selection for PSM Level FDR Calculation Based on Score: Automatic

- Reset Confidences for Nodes without Decoy Search (Fixed score thresholds): False

------------------------------------------------------------------

Processing node 3: Peptide and Protein Filter

------------------------------------------------------------------

1. Peptide Filters:

- Peptide Confidence At Least: High

- Keep Lower Confident PSMs: False

- Minimum Peptide Length: 6

- Remove Peptides Without Protein Reference: False

2. Protein Filters:

- Minimum Number of Peptide Sequences: 1

- Count Only Rank 1 Peptides: False

- Count Peptides Only for Top Scored Protein: False

------------------------------------------------------------------

Processing node 4: Protein Scorer

------------------------------------------------------------------

No parameters

------------------------------------------------------------------

Processing node 7: Protein FDR Validator

------------------------------------------------------------------

1. Confidence Thresholds:

- Target FDR (Strict): 0.01

- Target FDR (Relaxed): 0.05

------------------------------------------------------------------

Processing node 5: Protein Grouping

------------------------------------------------------------------

1. Protein Grouping:

- Apply strict parsimony principle: True

------------------------------------------------------------------

Processing node 6: Peptide in Protein Annotation

------------------------------------------------------------------

1. Flanking Residues:

- Annotate Flanking Residues of the Peptide: True

- Number Flanking Residues in Connection Tables: 1

2. Modifications in Peptide:

- Protein Modifications Reported: Only for Master Proteins

3. Modifications in Protein:

- Modification Sites Reported: All And Specific

- Minimum PSM Confidence: High

- Report Only PTMs: True

4. Positions in Protein:

- Protein Positions for Peptides: Only for Master Proteins

------------------------------------------------------------------

Processing node 9: Protein Marker

------------------------------------------------------------------

5. Annotate Species:

- As Species Map: False

- As Species Names: False

6. Mark Additional Entities:

- Annotation Groups: False

- Pathway Groups: False

- Modification Sites: True

- Peptide Isoform Groups: True

------------------------------------------------------------------

Processing node 8: Protein Annotation

------------------------------------------------------------------

1. Annotation Aspects:

- 1. Aspect: Biological Process

- 2. Aspect: Cellular Component

- 3. Aspect: Molecular Function

- 4. Aspect: None

- 5. Aspect: None

- 6. Aspect: None

2. Annotation/Pathway Groups:

- Protein Database: Uniprot_20230503.fasta

------------------------------------------------------------------

Processing node 10: Result Statistics

------------------------------------------------------------------

No parameters

------------------------------------------------------------------

Processing node 11: Display Settings

------------------------------------------------------------------

1. General:

- Filter Set:

\###	Filter Set MasterProteinFilter.filterset contains the following filters:

\###	Row Filter for TargetProtein:

\###	Master is equal to Master

\###

'magellan filter set' 1 'MasterProteinFilter.filterset' FiltersetProperties 1 'LastFileName' 'C:\Users\frank.berg\Desktop\MasterProteinFilter.filterset' Filter 'TargetProtein' 1 NARY_AND 1 = FilterConditionProperties 1 'NamedComparableFilterCondition/DisplayPropertyHint' 'Master' property 'Thermo.PD.EntityDataFramework.MasterProteinAssessment, Thermo.Magellan.EntityDataFramework' 'IsMasterProtein' constant 'Thermo.PD.EntityDataFramework.MasterProteinAssessment, Thermo.Magellan.EntityDataFramework' 'IsMasterProtein'

------------------------------------------------------------------

Processing node 12: Data Distributions

------------------------------------------------------------------

1. ID Distributions (Bottom-up):

- Peptides to Use: Only unique peptides based on protein groups

------------------------------------------------------------------

Workflow messages:

------------------------------------------------------------------

09/07/2023 08:32 AM	Job Execution:	Processing D:\P1266_QE_FY2024\P1290\P1290-01_M4_WT-(2).pdResult

09/07/2023 08:32 AM	(0) MSF Files:	D:\P1266_QE_FY2024\P1290\P1290-01_M4_WT-(2).msf

09/07/2023 08:32 AM	(0) MSF Files:	All 1 files are ready for processing.

09/07/2023 08:32 AM	(0) MSF Files:	Start transferring results of 1 msf files...

09/07/2023 08:32 AM	(0) MSF Files:	Transferred 38228 Proteins to result file in 11.3 s.

09/07/2023 08:32 AM	(0) MSF Files:	Transferred 33849 Decoy Proteins to result file in 10.1 s.

09/07/2023 08:32 AM	(0) MSF Files:	Added 16460 Mass Spectra to result file.

09/07/2023 08:32 AM	(0) MSF Files:	Added 2 traces to result file.

09/07/2023 08:32 AM	(0) MSF Files:	Added 1 Input Files to result file.

09/07/2023 08:33 AM	(0) MSF Files:	Added 20142 PSMs to result file.

09/07/2023 08:33 AM	(0) MSF Files:	Added 13557 Decoy PSMs to result file.

09/07/2023 08:33 AM	(0) MSF Files:	Added 18202 MS/MS Spectrum Info to result file.

09/07/2023 08:33 AM	(0) MSF Files:	Added 26915 Precursors to result file.

09/07/2023 08:33 AM	(0) MSF Files:	Added 1 Mass Recalibrations to result file.

09/07/2023 08:33 AM	(0) MSF Files:	Added 1318 Correction Landmarks to result file.

09/07/2023 08:33 AM	(0) MSF Files:	Added 2 Specialized Traces to result file.

09/07/2023 08:33 AM	(0) MSF Files:	Copying data took 37.8 s.

09/07/2023 08:33 AM	(0) MSF Files:	Finding unique sequences took 2.2 s.

09/07/2023 08:33 AM	(0) MSF Files:	Filtering proteins took 1.3 s.

09/07/2023 08:33 AM	(0) MSF Files:	-- Total execution of MSF Files (0) took 56 s --

09/07/2023 08:33 AM	(1) PSM Grouper:	Grouping 'PSMs'

09/07/2023 08:33 AM	(1) PSM Grouper:	Found 9762 Peptide Groups.

09/07/2023 08:33 AM	(1) PSM Grouper:	Grouping 'Decoy PSMs'

09/07/2023 08:34 AM	(1) PSM Grouper:	Found 7901 Decoy Peptide Groups.

09/07/2023 08:34 AM	(1) PSM Grouper:	-- Total execution of PSM Grouper (1) took 34.1 s --

09/07/2023 08:34 AM	(2) Peptide Validator:	Start PSM and Peptide validation in 'Automatic (Control peptide level error rate if possible)' mode...

09/07/2023 08:34 AM	(2) Peptide Validator:	Updated PSM confidences in 694.4 ms.

09/07/2023 08:34 AM	(2) Peptide Validator:	Use svm score for 'peptide' qvality run.

09/07/2023 08:34 AM	(2) Peptide Validator:	Updated peptide confidences using qvality in 5.1 s.

09/07/2023 08:34 AM	(2) Peptide Validator:	Calculating Mascot thresholds.

09/07/2023 08:34 AM	(2) Peptide Validator:	-- Total execution of Peptide Validator (2) took 6.4 s --

09/07/2023 08:34 AM	(3) Peptide and Protein Filter:	Filter Peptide Groups

09/07/2023 08:34 AM	(3) Peptide and Protein Filter:	Filter 1314/9762 Peptide Groups (8448 excluded) and 5186/20142 PSMs (14956 excluded) took 1.1 s

09/07/2023 08:34 AM	(3) Peptide and Protein Filter:	Filtering Proteins

09/07/2023 08:34 AM	(3) Peptide and Protein Filter:	Filter 3255/38228 Proteins (34973 excluded) took 1.4 s

09/07/2023 08:34 AM	(3) Peptide and Protein Filter:	Filter Decoy Peptide Groups

09/07/2023 08:34 AM	(3) Peptide and Protein Filter:	Filter 11/7901 Decoy Peptide Groups (7890 excluded) and 15/13557 Decoy PSMs (13542 excluded) took 700.9 ms

09/07/2023 08:34 AM	(3) Peptide and Protein Filter:	Filtering Decoy Proteins

09/07/2023 08:34 AM	(3) Peptide and Protein Filter:	Filter 24/33849 Decoy Proteins (33825 excluded) took 989.1 ms

09/07/2023 08:34 AM	(3) Peptide and Protein Filter:	Updating counts took 9.9 s.

09/07/2023 08:34 AM	(3) Peptide and Protein Filter:	-- Total execution of Peptide and Protein Filter (3) took 14.7 s --

09/07/2023 08:34 AM	(4) Protein Scorer:	Calculating Coverage and Counts

09/07/2023 08:34 AM	(4) Protein Scorer:	Calculated counts and coverages in 1.7 s.

09/07/2023 08:34 AM	(4) Protein Scorer:	Scoring target proteins

09/07/2023 08:34 AM	(4) Protein Scorer:	Scored 3255 proteins in 4.7 s.

09/07/2023 08:34 AM	(4) Protein Scorer:	Scoring decoy proteins

09/07/2023 08:34 AM	(4) Protein Scorer:	Scored 24 decoy proteins in 163.2 ms.

09/07/2023 08:34 AM	(4) Protein Scorer:	-- Total execution of Protein Scorer (4) took 6.7 s --

09/07/2023 08:34 AM	(7) Protein FDR Validator:	Apply confidences based on protein scores of 3255 proteins.

09/07/2023 08:34 AM	(7) Protein FDR Validator:	Calculating Sum PEP Score for Proteins

09/07/2023 08:34 AM	(7) Protein FDR Validator:	Calculating Sum PEP Score for Decoy Proteins

09/07/2023 08:34 AM	(7) Protein FDR Validator:	-- Total execution of Protein FDR Validator (7) took 2.2 s --

09/07/2023 08:34 AM	(5) Protein Grouping:	Retrieving 335 protein groups took 93.2 ms.

09/07/2023 08:34 AM	(5) Protein Grouping:	Storing, updating and connecting protein groups, PSMs and peptides took 8.1 s.

09/07/2023 08:34 AM	(5) Protein Grouping:	Applying strict parsimony for 335 protein groups.

09/07/2023 08:34 AM	(5) Protein Grouping:	Applying strict parsimony took 5.6 s.

09/07/2023 08:34 AM	(5) Protein Grouping:	Found 331 protein groups.

09/07/2023 08:34 AM	(5) Protein Grouping:	Retrieving 11 decoy protein groups took 20 ms.

09/07/2023 08:34 AM	(5) Protein Grouping:	Storing, updating and connecting protein groups, PSMs and peptides took 3.6 s.

09/07/2023 08:34 AM	(5) Protein Grouping:	Found 11 decoy protein groups.

09/07/2023 08:34 AM	(5) Protein Grouping:	-- Total execution of Protein Grouping (5) took 19.9 s --

09/07/2023 08:34 AM	(6) Peptide in Protein Annotation:	Start retrieving flanking residues and positions...

09/07/2023 08:35 AM	(6) Peptide in Protein Annotation:	Annotated PSMs/peptides for 3255 proteins.

09/07/2023 08:35 AM	(6) Peptide in Protein Annotation:	Start annotating peptide groups with flanking residues and positions...

09/07/2023 08:35 AM	(6) Peptide in Protein Annotation:	Annotate Peptide Groups items

09/07/2023 08:35 AM	(6) Peptide in Protein Annotation:	Start calculating protein coverage...

09/07/2023 08:35 AM	(6) Peptide in Protein Annotation:	-- Total execution of Peptide in Protein Annotation (6) took 52.8 s --

09/07/2023 08:35 AM	(8) Protein Annotation:	Using annotation server https://proteinannotationservice.thermofisher.com/.

09/07/2023 08:35 AM	(8) Protein Annotation:	Protein annotation might take significantly long time if outdated or on-disk FASTA files are used.

09/07/2023 08:35 AM	(8) Protein Annotation:	Save annotations of Uniprot_20230503.fasta in result file...

09/07/2023 08:41 AM	(8) Protein Annotation:	Start saving annotation descriptions in result file

09/07/2023 08:41 AM	(8) Protein Annotation:	Retrieving annotations from the Proteome Discoverer protein knowledge-base.

09/07/2023 08:41 AM	(8) Protein Annotation:	No chromosome information available, no "Chromosome" column created.

09/07/2023 08:41 AM	(8) Protein Annotation:	Found annotations for 3255 of 3255 proteins.

09/07/2023 08:41 AM	(8) Protein Annotation:	Found 5411 annotation groups.

09/07/2023 08:41 AM	(8) Protein Annotation:	Found 824 pathway groups.

09/07/2023 08:41 AM	(8) Protein Annotation:	Calculating associated data for annotation and pathway groups.

09/07/2023 08:42 AM	(8) Protein Annotation:	Preparing the annotation and pathway groups for result file

09/07/2023 08:42 AM	(8) Protein Annotation:	Discrepancies found in 23 annotation groups (# Total Proteins < # Identified Proteins!). To get accurate and non-conflicting annotation results it is recommended to use the same protein database(s) used in the search node(s) to annotate the annotation groups.

09/07/2023 08:42 AM	(8) Protein Annotation:	Discrepancies found in 22 pathway groups (# Total Proteins < # Identified Proteins!). To get accurate and non-conflicting annotation results it is recommended to use the same protein database(s) used in the search node(s) to annotate the pathway groups.

09/07/2023 08:42 AM	(8) Protein Annotation:	-- Total execution of Protein Annotation (8) took 6 min 29 s --

09/07/2023 08:42 AM	(9) Protein Marker:	Proteins will not be marked, because no contaminant file or FASTA file is specified.

09/07/2023 08:42 AM	(9) Protein Marker:	Contaminants Detection took: 7.2 ms

09/07/2023 08:42 AM	(9) Protein Marker:	Annotating species names took: 9.2 ms

09/07/2023 08:42 AM	(9) Protein Marker:	-- Total execution of Protein Marker (9) took 23.6 ms --

09/07/2023 08:42 AM	(10) Result Statistics:	-- Total execution of Result Statistics (10) took 772 ms --

09/07/2023 08:42 AM	(11) Display Settings:	Applying display filter and layout

09/07/2023 08:42 AM	(11) Display Settings:	-- Total execution of Display Settings (11) took 46.9 ms --

09/07/2023 08:42 AM	(12) Data Distributions:	Calculating found for samples

09/07/2023 08:42 AM	(12) Data Distributions:	Calculating found for sample groups

09/07/2023 08:42 AM	(12) Data Distributions:	-- Total execution of Data Distributions (12) took 3.2 s --

09/07/2023 08:42 AM	Job Execution:	----- Total Job execution took: 9 min 46 s. -----

09/07/2023 08:42 AM	Job Execution:	Finalizing file took 18.7 s.

09/07/2023 08:42 AM	Job Execution:	Finished D:\P1266_QE_FY2024\P1290\P1290-01_M4_WT-(2).pdResult

================================================================================

Processing Step A: Workflow

================================================================================

Result name: P1290-01_M4_WT

Result file: D:\P1266_QE_FY2024\P1290\P1290-01_M4_WT-(2).msf

Description: Protein ID with Masco and Sequest HT Uniprott-Homo sapiens

Workflow based on template: ProteinID_Uniprot-Homo_sapiens

Creation date: 9/7/2023 7:17:58 AM

Created with Discoverer version: 3.0.0.757

------------------------------------------------------------------

The workflow tree:

------------------------------------------------------------------

|-(11) Spectrum Files RC

|-(8) Spectrum Selector

|-(9) Precursor Detector

|-(12) Sequest HT

|-(6) Percolator

|-(13) Mascot

|-(7) Percolator

------------------------------------------------------------------

Processing node 11: Spectrum Files RC

------------------------------------------------------------------

1. Search Settings:

- File Name(s): D:\P1266_QE_FY2024\P1290\P1290-01_M4_WT.raw

- Protein Database: Uniprot-Homo_sapiens.fasta

- Enzyme Name: Trypsin (Full)

- Precursor Mass Tolerance: 20 ppm

- Fragment Mass Tolerance: 0.5 Da

- 1. Dynamic Modification: Oxidation / +15.995 Da (M)

- 1. Static Modification: Carbamidomethyl / +57.021 Da (C)

2. Regression Settings:

- Regression Model: Non-linear Regression

- Parameter Tuning: Coarse

------------------------------------------------------------------

Processing node 8: Spectrum Selector

------------------------------------------------------------------

1. General Settings:

- Precursor Selection: Use MS1 Precursor

- Use Isotope Pattern in Precursor Reevaluation: True

- Provide Profile Spectra: Automatic

2. Spectrum Properties Filter:

- Lower RT Limit: 0

- Upper RT Limit: 0

- First Scan: 0

- Last Scan: 0

- Lowest Charge State: 0

- Highest Charge State: 0

- Min. Precursor Mass: 350 Da

- Max. Precursor Mass: 5000 Da

- Total Intensity Threshold: 0

- Minimum Peak Count: 1

3. Scan Event Filters:

- MS Order: Is Not MS1

- Min. Collision Energy: 0

- Max. Collision Energy: 1000

- Scan Type: Is Full

4. Peak Filters:

- S/N Threshold (FT-only): 1.5

5. Replacements for Unrecognized Properties:

- Unrecognized Charge Replacements: Automatic

- Unrecognized Mass Analyzer Replacements: ITMS

- Unrecognized MS Order Replacements: MS2

- Unrecognized Activation Type Replacements: CID

- Unrecognized Polarity Replacements: +

- Unrecognized MS Resolution@200 Replacements: 60000

- Unrecognized MSn Resolution@200 Replacements: 30000

6. Precursor Pattern Extraction:

- Precursor Clipping Range Before: 2.5 Da

- Precursor Clipping Range After: 5.5 Da

------------------------------------------------------------------

Processing node 9: Precursor Detector

------------------------------------------------------------------

1. General Settings:

- S/N Threshold: 1.5

- S/N Threshold for most abundant peak: 1.5

------------------------------------------------------------------

Processing node 12: Sequest HT

------------------------------------------------------------------

1. Input Data:

- Protein Database: Uniprot-Homo_sapiens.fasta

- Enzyme Name: Trypsin (Full)

- Max. Missed Cleavage Sites: 2

- Min. Peptide Length: 6

- Max. Peptide Length: 150

- Max. Number of Peptides Reported: 10

2. Tolerances:

- Precursor Mass Tolerance: 10 ppm

- Fragment Mass Tolerance: 0.02 Da

- Use Average Precursor Mass: False

- Use Average Fragment Mass: False

3. Spectrum Matching:

- Use Neutral Loss a Ions: True

- Use Neutral Loss b Ions: True

- Use Neutral Loss y Ions: True

- Use Flanking Ions: True

- Weight of a Ions: 0

- Weight of b Ions: 1

- Weight of c Ions: 0

- Weight of x Ions: 0

- Weight of y Ions: 1

- Weight of z Ions: 0

4. Dynamic Modifications:

- Max. Equal Modifications Per Peptide: 3

- Max. Dynamic Modifications Per Peptide: 4

- 1. Dynamic Modification: Oxidation / +15.995 Da (M)

- 2. Dynamic Modification: Deamidated / +0.984 Da (N, Q)

6. Dynamic Modifications (protein terminus):

- 1. N-Terminal Modification: Acetyl / +42.011 Da (N-Terminus)

7. Static Modifications:

- 1. Static Modification: Carbamidomethyl / +57.021 Da (C)

------------------------------------------------------------------

Processing node 6: Percolator

------------------------------------------------------------------

1. Target/Decoy Strategy:

- Target/Decoy Selection: Concatenated

- Validation based on: q-Value

2. Input Data:

- Maximum Delta Cn: 0.05

- Maximum Rank: 0

3. FDR Targets:

- Target FDR (Strict): 0.01

- Target FDR (Relaxed): 0.05

------------------------------------------------------------------

Processing node 13: Mascot

------------------------------------------------------------------

1. Input Data:

- Instrument: Default

- Protein Database: Uniprot-Homo_sapiens

- Enzyme Name: Trypsin

- Maximum Missed Cleavage Sites: 1

- Taxonomy: All entries

- Error Tolerant Search: False

2. Tolerances:

- Fragment Mass Tolerance: 0.02 Da

- Precursor Mass Tolerance: 10 ppm

- Use Average Precursor Mass: False

4. Dynamic Modifications:

- Show All Modifications: False

- 1. Dynamic Modification: Oxidation (M)

- 2. Dynamic Modification: Deamidated (NQ)

- 3. Dynamic Modification: Acetyl (Protein N-term)

5. Static Modifications:

- 1. Static Modification: Carbamidomethyl (C)

------------------------------------------------------------------

Processing node 7: Percolator

------------------------------------------------------------------

1. Target/Decoy Strategy:

- Target/Decoy Selection: Concatenated

- Validation based on: q-Value

2. Input Data:

- Maximum Delta Cn: 0.05

- Maximum Rank: 0

3. FDR Targets:

- Target FDR (Strict): 0.01

- Target FDR (Relaxed): 0.05

------------------------------------------------------------------

Workflow messages:

------------------------------------------------------------------

09/07/2023 07:17 AM	Job Execution:	Processing D:\P1266_QE_FY2024\P1290\P1290-01_M4_WT-(2).msf

09/07/2023 07:18 AM	(11) Spectrum Files RC:	Start processing file F1: P1290-01_M4_WT.raw...

09/07/2023 07:18 AM	(11) Spectrum Files RC:	Retrieving 18232 spectra took 47.4 s

09/07/2023 07:18 AM	(11) Spectrum Files RC:	There is already an adequate target FASTA index for Uniprot-Homo_sapiens(2169adde-9cc1-4003-8cd1-ef6f92ad5805).fasta.

09/07/2023 07:18 AM	(11) Spectrum Files RC:	Start searching spectra (HCD (High Energy Collision Dissociation))...

09/07/2023 07:18 AM	(11) Spectrum Files RC:	ISE (2.0.0.26, x64) started at 9/7/2023 7:18:49 AM on MASCOTSERVER (x64) [32 CPUs] running Microsoft Windows NT 6.2.9200.0 (64bit) [.NET: 4.0.30319.42000]

09/07/2023 07:18 AM	(11) Spectrum Files RC:	Workload level: #parallel tasks: 10

09/07/2023 07:18 AM	(11) Spectrum Files RC:	Workload level: #spectra loaded and processed at once: 10000

09/07/2023 07:18 AM	(11) Spectrum Files RC:	On-Disk search is performed

09/07/2023 07:19 AM	(11) Spectrum Files RC:	Average search time per spectrum was 1.1 ms.

09/07/2023 07:19 AM	(11) Spectrum Files RC:	Start reading spectrum results...

09/07/2023 07:19 AM	(11) Spectrum Files RC:	Start calculating calibration...

09/07/2023 07:19 AM	(11) Spectrum Files RC:	Processing file F1 took 1 min 15 s.

09/07/2023 07:19 AM	(11) Spectrum Files RC:	-- Total execution of Spectrum Files RC (11) took 1 min 15 s --

09/07/2023 07:19 AM	(8) Spectrum Selector:	Profile spectra are not sent.

09/07/2023 07:19 AM	(8) Spectrum Selector:	Reading from file 1 of 1 F1: D:\P1266_QE_FY2024\P1290\P1290-01_M4_WT.raw (20541 spectra total)

09/07/2023 07:19 AM	(12) Sequest HT:	Sequence Database: Uniprot-Homo_sapiens.fasta

09/07/2023 07:19 AM	(13) Mascot:	Use mascot server http://localhost/mascot/ with Mascot version 2.8.2

09/07/2023 07:19 AM	(13) Mascot:	Sequence Database: Uniprot-Homo_sapiens

09/07/2023 07:20 AM	(8) Spectrum Selector:	Sent 18202 spectra from file F1.

09/07/2023 07:20 AM	(8) Spectrum Selector:	Sent 18202 spectra from 1 files (processing time: 41 s).

09/07/2023 07:20 AM	(8) Spectrum Selector:	-- Total execution of Spectrum Selector (8) took 1 min 25 s --

09/07/2023 07:20 AM	(9) Precursor Detector:	-- Total execution of Precursor Detector (9) took 10 s --

09/07/2023 07:20 AM	(12) Sequest HT:	Storing spectra took 25.6 s.

09/07/2023 07:20 AM	(12) Sequest HT:	There is already an adequate target FASTA index for Uniprot-Homo_sapiens(2169adde-9cc1-4003-8cd1-ef6f92ad5805).fasta.

09/07/2023 07:20 AM	(12) Sequest HT:	Start Sequest HT target search for 18202 spectra (26915 precursors)...

09/07/2023 07:20 AM	(12) Sequest HT:	ISE (2.0.0.26, x64) started at 9/7/2023 7:20:43 AM on MASCOTSERVER (x64) [32 CPUs] running Microsoft Windows NT 6.2.9200.0 (64bit) [.NET: 4.0.30319.42000]

09/07/2023 07:20 AM	(12) Sequest HT:	Workload level: #parallel tasks: 10

09/07/2023 07:20 AM	(12) Sequest HT:	Workload level: #spectra loaded and processed at once: 10000

09/07/2023 07:20 AM	(12) Sequest HT:	On-Disk search is performed

09/07/2023 07:23 AM	(12) Sequest HT:	27 spectra were not used for search.

09/07/2023 07:23 AM	(12) Sequest HT:	Average search time per spectrum was 7.0 ms.

09/07/2023 07:23 AM	(12) Sequest HT:	Performing target search took 3 min 11 s.

09/07/2023 07:24 AM	(12) Sequest HT:	Stored 44803 PSMs for 26888 spectra

09/07/2023 07:24 AM	(12) Sequest HT:	Discarded 69999 peptide(s) that did not match the conditions for protein terminal modifications.

09/07/2023 07:24 AM	(12) Sequest HT:	Reading search results took 19.4 s.

09/07/2023 07:24 AM	(12) Sequest HT:	Saving results took 6.4 s.

09/07/2023 07:24 AM	(12) Sequest HT:	Saving proteins took 29.2 s.

09/07/2023 07:24 AM	(12) Sequest HT:	Reading results took 55.4 s.

09/07/2023 07:24 AM	(12) Sequest HT:	There is already an adequate decoy FASTA index for Uniprot-Homo_sapiens(2169adde-9cc1-4003-8cd1-ef6f92ad5805)_reversed.fasta.

09/07/2023 07:24 AM	(12) Sequest HT:	Start Sequest HT decoy search for 18202 spectra (26915 precursors)...

09/07/2023 07:24 AM	(12) Sequest HT:	ISE (2.0.0.26, x64) started at 9/7/2023 7:24:49 AM on MASCOTSERVER (x64) [32 CPUs] running Microsoft Windows NT 6.2.9200.0 (64bit) [.NET: 4.0.30319.42000]

09/07/2023 07:24 AM	(12) Sequest HT:	Workload level: #parallel tasks: 10

09/07/2023 07:24 AM	(12) Sequest HT:	Workload level: #spectra loaded and processed at once: 10000

09/07/2023 07:24 AM	(12) Sequest HT:	On-Disk search is performed

09/07/2023 07:28 AM	(12) Sequest HT:	27 spectra were not used for search.

09/07/2023 07:28 AM	(12) Sequest HT:	Average search time per spectrum was 7.3 ms.

09/07/2023 07:28 AM	(12) Sequest HT:	Performing decoy search took 3 min 19 s.

09/07/2023 07:28 AM	(12) Sequest HT:	Stored 48683 decoy PSMs for 26888 spectra

09/07/2023 07:28 AM	(12) Sequest HT:	Discarded 69899 peptide(s) that did not match the conditions for protein terminal modifications.

09/07/2023 07:28 AM	(12) Sequest HT:	Reading search results took 16.2 s.

09/07/2023 07:28 AM	(12) Sequest HT:	Saving results took 6.2 s.

09/07/2023 07:28 AM	(12) Sequest HT:	Saving proteins took 27.1 s.

09/07/2023 07:28 AM	(12) Sequest HT:	Reading results took 49.7 s.

09/07/2023 07:28 AM	(12) Sequest HT:	Finalizing search results...

09/07/2023 07:29 AM	(12) Sequest HT:	-- Total execution of Sequest HT (12) took 8 min 17 s --

09/07/2023 07:30 AM	(6) Percolator:	Creating percolator input file with results from Sequest HT (12) took 55.5 s.

09/07/2023 07:30 AM	(6) Percolator:	The input file contains 25892 PSMs, 24094 decoy PSMs and 32 features.

09/07/2023 07:30 AM	(6) Percolator:	Generated percolator input for concatenated mode

09/07/2023 07:30 AM	(6) Percolator:	Percolator version 3.05.0, Build Date May 18 2020 02:35:01

09/07/2023 07:30 AM	(6) Percolator:	Copyright (c) 2006-9 University of Washington. All rights reserved.

09/07/2023 07:30 AM	(6) Percolator:	Written by Lukas K├ñll (lukall@u.washington.edu) in the

09/07/2023 07:30 AM	(6) Percolator:	Department of Genome Sciences at the University of Washington.

09/07/2023 07:30 AM	(6) Percolator:	Issued command:

09/07/2023 07:30 AM	(6) Percolator:	C:\Program Files\Thermo\Proteome Discoverer 3.0\Proteome Discoverer 3.0\Tools\Percolator\win\percolator.exe --num-threads 18 -s -X C:\ProgramData\Thermo\Proteome Discoverer 3.0\Scratch\Job3185\Percol(6)\output12.xml -N 0 -Z -Y C:\ProgramData\Thermo\Proteome Discoverer 3.0\Scratch\Job3185\Percol(6)\input12.tab

09/07/2023 07:30 AM	(6) Percolator:	Started Thu Sep 7 07:30:10 2023

09/07/2023 07:30 AM	(6) Percolator:	Hyperparameters: selectionFdr=0.01, Cpos=0, Cneg=0, maxNiter=10

09/07/2023 07:30 AM	(6) Percolator:	Reading tab-delimited input from datafile C:\ProgramData\Thermo\Proteome Discoverer 3.0\Scratch\Job3185\Percol(6)\input12.tab

09/07/2023 07:30 AM	(6) Percolator:	Features:

09/07/2023 07:30 AM	(6) Percolator:	XCorr Delta Cn From Second PSM Binomial Score Isolation Interference [%] MH+ [Da] Delta Mass [Da] Delta Mass [ppm] Absolute Delta Mass [Da] Absolute Delta Mass [ppm] Peptide Length Is z=1 Is z=2 Is z=3 Is z=4 Is z=5 Is z>5 # Missed Cleavages Log Peptides Matched Log Total Intensity Fraction Matched Intensity [%] Fragment Coverage Series A, B, C [%] Fragment Coverage Series X, Y, Z [%] Log Matched Fragment Series Intensities A, B, C Log Matched Fragment Series Intensities X, Y, Z Longest Sequence Series A, B, C Longest Sequence Series X, Y, Z IQR Fragment Delta Mass [Da] IQR Fragment Delta Mass [ppm] Mean Fragment Delta Mass [Da] Mean Fragment Delta Mass [ppm] Mean Absolute Fragment Delta Mass [Da] Mean Absolute Fragment Delta Mass [ppm]

09/07/2023 07:30 AM	(6) Percolator:	Found 49986 PSMs

09/07/2023 07:30 AM	(6) Percolator:	Separate target and decoy search inputs detected, using target-decoy competition on Percolator scores.

09/07/2023 07:30 AM	(6) Percolator:	Train/test set contains 25892 positives and 24094 negatives, size ratio=1.07462 and pi0=1

09/07/2023 07:30 AM	(6) Percolator:	Selecting Cpos by cross-validation.

09/07/2023 07:30 AM	(6) Percolator:	Selecting Cneg by cross-validation.

09/07/2023 07:30 AM	(6) Percolator:	Split 1:	Selected feature 3 as initial direction. Could separate 1069 training set positives with q<0.01 in that direction.

09/07/2023 07:30 AM	(6) Percolator:	Split 2:	Selected feature 3 as initial direction. Could separate 1071 training set positives with q<0.01 in that direction.

09/07/2023 07:30 AM	(6) Percolator:	Split 3:	Selected feature 3 as initial direction. Could separate 1133 training set positives with q<0.01 in that direction.

09/07/2023 07:30 AM	(6) Percolator:	Found 1577 test set positives with q<0.01 in initial direction

09/07/2023 07:30 AM	(6) Percolator:	Reading in data and feature calculation took 1.72 cpu seconds or 1 seconds wall clock time.

09/07/2023 07:30 AM	(6) Percolator:	---Training with Cpos selected by cross validation, Cneg selected by cross validation, initial_fdr=0.01, fdr=0.01

09/07/2023 07:30 AM	(6) Percolator:	Iteration 1:	Estimated 2677 PSMs with q<0.01

09/07/2023 07:30 AM	(6) Percolator:	Iteration 2:	Estimated 2814 PSMs with q<0.01

09/07/2023 07:30 AM	(6) Percolator:	Iteration 3:	Estimated 2899 PSMs with q<0.01

09/07/2023 07:30 AM	(6) Percolator:	Iteration 4:	Estimated 2944 PSMs with q<0.01

09/07/2023 07:30 AM	(6) Percolator:	Iteration 5:	Estimated 2965 PSMs with q<0.01

09/07/2023 07:30 AM	(6) Percolator:	Iteration 6:	Estimated 2984 PSMs with q<0.01

09/07/2023 07:30 AM	(6) Percolator:	Iteration 7:	Estimated 3008 PSMs with q<0.01

09/07/2023 07:30 AM	(6) Percolator:	Iteration 8:	Estimated 3018 PSMs with q<0.01

09/07/2023 07:30 AM	(6) Percolator:	Iteration 9:	Estimated 3013 PSMs with q<0.01

09/07/2023 07:30 AM	(6) Percolator:	Iteration 10:	Estimated 3029 PSMs with q<0.01

09/07/2023 07:30 AM	(6) Percolator:	Learned normalized SVM weights for the 3 cross-validation splits:

09/07/2023 07:30 AM	(6) Percolator:	Split1	Split2	Split3	FeatureName

09/07/2023 07:30 AM	(6) Percolator:	-0.1214	-0.5845	0.3962	XCorr

09/07/2023 07:30 AM	(6) Percolator:	1.1966	0.9872	0.1197	Delta Cn From Second PSM

09/07/2023 07:30 AM	(6) Percolator:	4.7708	5.2485	7.4426	Binomial Score

09/07/2023 07:30 AM	(6) Percolator:	-0.1218	0.0757	0.6391	Isolation Interference [%]

09/07/2023 07:30 AM	(6) Percolator:	-0.3237	-0.4200	0.7351	MH+ [Da]

09/07/2023 07:30 AM	(6) Percolator:	0.6596	0.3378	-0.9670	Delta Mass [Da]

09/07/2023 07:30 AM	(6) Percolator:	-0.1097	-0.1460	0.6200	Delta Mass [ppm]

09/07/2023 07:30 AM	(6) Percolator:	-1.3003	-0.5487	-0.9278	Absolute Delta Mass [Da]

09/07/2023 07:30 AM	(6) Percolator:	0.3047	-0.2572	0.0000	Absolute Delta Mass [ppm]

09/07/2023 07:30 AM	(6) Percolator:	-1.2616	-0.2188	-1.6587	Peptide Length

09/07/2023 07:30 AM	(6) Percolator:	-0.4192	-0.6960	-0.4332	Is z=1

09/07/2023 07:30 AM	(6) Percolator:	-0.1258	0.1870	0.3300	Is z=2

09/07/2023 07:30 AM	(6) Percolator:	0.2982	0.3246	-0.1399	Is z=3

09/07/2023 07:30 AM	(6) Percolator:	0.6619	0.3247	0.1712	Is z=4

09/07/2023 07:30 AM	(6) Percolator:	0.2024	0.0727	0.3219	Is z=5

09/07/2023 07:30 AM	(6) Percolator:	0.1179	0.0605	0.1331	Is z>5

09/07/2023 07:30 AM	(6) Percolator:	-0.5739	-0.7231	-0.8954	# Missed Cleavages

09/07/2023 07:30 AM	(6) Percolator:	0.0251	-0.2496	-0.2845	Log Peptides Matched

09/07/2023 07:30 AM	(6) Percolator:	-1.4882	-1.2749	-3.1346	Log Total Intensity

09/07/2023 07:30 AM	(6) Percolator:	-0.2360	-0.3618	-0.5276	Fraction Matched Intensity [%]

09/07/2023 07:30 AM	(6) Percolator:	-1.5805	-0.9701	-1.5945	Fragment Coverage Series A, B, C [%]

09/07/2023 07:30 AM	(6) Percolator:	-0.4792	-0.7276	-0.8875	Fragment Coverage Series X, Y, Z [%]

09/07/2023 07:30 AM	(6) Percolator:	0.0420	-0.1278	0.3492	Log Matched Fragment Series Intensities A, B, C

09/07/2023 07:30 AM	(6) Percolator:	-0.8321	-0.7546	0.3650	Log Matched Fragment Series Intensities X, Y, Z

09/07/2023 07:30 AM	(6) Percolator:	0.0750	-0.4848	-0.9895	Longest Sequence Series A, B, C

09/07/2023 07:30 AM	(6) Percolator:	-0.0469	0.0777	0.2303	Longest Sequence Series X, Y, Z

09/07/2023 07:30 AM	(6) Percolator:	-0.1753	-0.2543	-0.3809	IQR Fragment Delta Mass [Da]

09/07/2023 07:30 AM	(6) Percolator:	-1.3773	0.4361	-1.7740	IQR Fragment Delta Mass [ppm]

09/07/2023 07:30 AM	(6) Percolator:	0.0476	-0.4150	-1.4485	Mean Fragment Delta Mass [Da]

09/07/2023 07:30 AM	(6) Percolator:	0.1759	0.4084	1.7877	Mean Fragment Delta Mass [ppm]

09/07/2023 07:30 AM	(6) Percolator:	-0.6523	-2.5277	-0.1861	Mean Absolute Fragment Delta Mass [Da]

09/07/2023 07:30 AM	(6) Percolator:	0.4154	1.1357	-1.4492	Mean Absolute Fragment Delta Mass [ppm]

09/07/2023 07:30 AM	(6) Percolator:	-5.8933	-6.2708	-9.6580	m0

09/07/2023 07:30 AM	(6) Percolator:	Found 2720 test set PSMs with q<0.01.

09/07/2023 07:30 AM	(6) Percolator:	Selected best-scoring PSM per scan+expMass (target-decoy competition): 12990 target PSMs and 9819 decoy PSMs.

09/07/2023 07:30 AM	(6) Percolator:	Tossing out "redundant" PSMs keeping only the best scoring PSM for each unique peptide.

09/07/2023 07:30 AM	(6) Percolator:	Calculating q values.

09/07/2023 07:30 AM	(6) Percolator:	Final list yields 1254 target peptides with q<0.01.

09/07/2023 07:30 AM	(6) Percolator:	Calculating posterior error probabilities (PEPs).

09/07/2023 07:30 AM	(6) Percolator:	Processing took 11.45 cpu seconds or 12 seconds wall clock time.

09/07/2023 07:30 AM	(6) Percolator:	PSMId	score	q-value	posterior_error_prob	peptide	proteinIds

09/07/2023 07:30 AM	(6) Percolator:	Percolator (64Bit) process execution took 14.3 s.

09/07/2023 07:30 AM	(6) Percolator:	2733/30 high confident target/decoy peptides were found for Sequest HT (12).

09/07/2023 07:30 AM	(6) Percolator:	506/136 medium confident target/decoy peptides were found for Sequest HT (12).

09/07/2023 07:30 AM	(6) Percolator:	-- Total execution of Percolator (64Bit) for Sequest HT (12) took 1 min 12 s --

09/07/2023 07:30 AM	(13) Mascot:	Storing spectra took 790.1 ms.

09/07/2023 07:30 AM	(13) Mascot:	Start searching 18202 spectra...

09/07/2023 07:30 AM	(13) Mascot:	Mascot result on server (filename=../data/20230907/F054556.dat)

09/07/2023 08:28 AM	(13) Mascot:	Mascot Server completed

09/07/2023 08:28 AM	(13) Mascot:	Received Mascot result file (filename=../data/20230907/F054556.dat)

09/07/2023 08:28 AM	(13) Mascot:	Start parsing 1 result files..

09/07/2023 08:28 AM	(13) Mascot:	Start parsing file 1 (50 MB)...

09/07/2023 08:28 AM	(13) Mascot:	Start mapping modifications...

09/07/2023 08:28 AM	(13) Mascot:	Start translating results...

09/07/2023 08:29 AM	(13) Mascot:	Received 26428 proteins from Mascot server (Uniprot-Homo_sapiens)

09/07/2023 08:29 AM	(13) Mascot:	Stored 10117 PSMs for 6009 spectra

09/07/2023 08:31 AM	(13) Mascot:	Received 33721 proteins from Mascot server (Uniprot-Homo_sapiens)

09/07/2023 08:31 AM	(13) Mascot:	Stored 10075 decoy PSMs for 4983 spectra

09/07/2023 08:31 AM	(13) Mascot:	Stored 26428 target and 33721 decoy proteins.

09/07/2023 08:31 AM	(13) Mascot:	Used mascot server http://localhost/mascot/ with Mascot version 2.8.2

09/07/2023 08:31 AM	(13) Mascot:	Finalizing search results...

09/07/2023 08:31 AM	(13) Mascot:	-- Total execution of Mascot (13) took 1 h 1 min --

09/07/2023 08:32 AM	(7) Percolator:	Creating percolator input file with results from Mascot (13) took 29.4 s.

09/07/2023 08:32 AM	(7) Percolator:	The input file contains 6441 PSMs, 3825 decoy PSMs and 32 features.

09/07/2023 08:32 AM	(7) Percolator:	Generated percolator input for concatenated mode

09/07/2023 08:32 AM	(7) Percolator:	Percolator version 3.05.0, Build Date May 18 2020 02:35:01

09/07/2023 08:32 AM	(7) Percolator:	Copyright (c) 2006-9 University of Washington. All rights reserved.

09/07/2023 08:32 AM	(7) Percolator:	Written by Lukas K├ñll (lukall@u.washington.edu) in the

09/07/2023 08:32 AM	(7) Percolator:	Department of Genome Sciences at the University of Washington.

09/07/2023 08:32 AM	(7) Percolator:	Issued command:

09/07/2023 08:32 AM	(7) Percolator:	C:\Program Files\Thermo\Proteome Discoverer 3.0\Proteome Discoverer 3.0\Tools\Percolator\win\percolator.exe --num-threads 18 -s -X C:\ProgramData\Thermo\Proteome Discoverer 3.0\Scratch\Job3185\Percol(7)\output13.xml -N 0 -Z -Y C:\ProgramData\Thermo\Proteome Discoverer 3.0\Scratch\Job3185\Percol(7)\input13.tab

09/07/2023 08:32 AM	(7) Percolator:	Started Thu Sep 7 08:32:22 2023

09/07/2023 08:32 AM	(7) Percolator:	Hyperparameters: selectionFdr=0.01, Cpos=0, Cneg=0, maxNiter=10

09/07/2023 08:32 AM	(7) Percolator:	Reading tab-delimited input from datafile C:\ProgramData\Thermo\Proteome Discoverer 3.0\Scratch\Job3185\Percol(7)\input13.tab

09/07/2023 08:32 AM	(7) Percolator:	Features:

09/07/2023 08:32 AM	(7) Percolator:	IonsScore Delta Cn From Second PSM Binomial Score Isolation Interference [%] MH+ [Da] Delta Mass [Da] Delta Mass [ppm] Absolute Delta Mass [Da] Absolute Delta Mass [ppm] Peptide Length Is z=1 Is z=2 Is z=3 Is z=4 Is z=5 Is z>5 # Missed Cleavages Log Peptides Matched Log Total Intensity Fraction Matched Intensity [%] Fragment Coverage Series A, B, C [%] Fragment Coverage Series X, Y, Z [%] Log Matched Fragment Series Intensities A, B, C Log Matched Fragment Series Intensities X, Y, Z Longest Sequence Series A, B, C Longest Sequence Series X, Y, Z IQR Fragment Delta Mass [Da] IQR Fragment Delta Mass [ppm] Mean Fragment Delta Mass [Da] Mean Fragment Delta Mass [ppm] Mean Absolute Fragment Delta Mass [Da] Mean Absolute Fragment Delta Mass [ppm]

09/07/2023 08:32 AM	(7) Percolator:	Found 10266 PSMs

09/07/2023 08:32 AM	(7) Percolator:	Separate target and decoy search inputs detected, using target-decoy competition on Percolator scores.

09/07/2023 08:32 AM	(7) Percolator:	Train/test set contains 6441 positives and 3825 negatives, size ratio=1.68392 and pi0=1

09/07/2023 08:32 AM	(7) Percolator:	Selecting Cpos by cross-validation.

09/07/2023 08:32 AM	(7) Percolator:	Selecting Cneg by cross-validation.

09/07/2023 08:32 AM	(7) Percolator:	Split 1:	Selected feature 1 as initial direction. Could separate 1303 training set positives with q<0.01 in that direction.

09/07/2023 08:32 AM	(7) Percolator:	Split 2:	Selected feature 1 as initial direction. Could separate 1321 training set positives with q<0.01 in that direction.

09/07/2023 08:32 AM	(7) Percolator:	Split 3:	Selected feature 1 as initial direction. Could separate 1415 training set positives with q<0.01 in that direction.

09/07/2023 08:32 AM	(7) Percolator:	Found 1876 test set positives with q<0.01 in initial direction

09/07/2023 08:32 AM	(7) Percolator:	Reading in data and feature calculation took 0.333 cpu seconds or 0 seconds wall clock time.

09/07/2023 08:32 AM	(7) Percolator:	---Training with Cpos selected by cross validation, Cneg selected by cross validation, initial_fdr=0.01, fdr=0.01

09/07/2023 08:32 AM	(7) Percolator:	Iteration 1:	Estimated 2609 PSMs with q<0.01

09/07/2023 08:32 AM	(7) Percolator:	Iteration 2:	Estimated 2708 PSMs with q<0.01

09/07/2023 08:32 AM	(7) Percolator:	Iteration 3:	Estimated 2743 PSMs with q<0.01

09/07/2023 08:32 AM	(7) Percolator:	Iteration 4:	Estimated 2752 PSMs with q<0.01

09/07/2023 08:32 AM	(7) Percolator:	Iteration 5:	Estimated 2771 PSMs with q<0.01

09/07/2023 08:32 AM	(7) Percolator:	Iteration 6:	Estimated 2781 PSMs with q<0.01

09/07/2023 08:32 AM	(7) Percolator:	Iteration 7:	Estimated 2780 PSMs with q<0.01

09/07/2023 08:32 AM	(7) Percolator:	Iteration 8:	Estimated 2782 PSMs with q<0.01

09/07/2023 08:32 AM	(7) Percolator:	Iteration 9:	Estimated 2781 PSMs with q<0.01

09/07/2023 08:32 AM	(7) Percolator:	Iteration 10:	Estimated 2780 PSMs with q<0.01

09/07/2023 08:32 AM	(7) Percolator:	Learned normalized SVM weights for the 3 cross-validation splits:

09/07/2023 08:32 AM	(7) Percolator:	Split1	Split2	Split3	FeatureName

09/07/2023 08:32 AM	(7) Percolator:	2.4311	2.3093	1.5581	IonsScore

09/07/2023 08:32 AM	(7) Percolator:	0.6287	0.7846	0.2906	Delta Cn From Second PSM

09/07/2023 08:32 AM	(7) Percolator:	3.5550	1.9542	2.3262	Binomial Score

09/07/2023 08:32 AM	(7) Percolator:	0.3168	0.2527	0.2663	Isolation Interference [%]

09/07/2023 08:32 AM	(7) Percolator:	-0.0693	-0.2743	-0.3336	MH+ [Da]

09/07/2023 08:32 AM	(7) Percolator:	0.2863	-0.2454	-0.6811	Delta Mass [Da]

09/07/2023 08:32 AM	(7) Percolator:	-0.2079	0.1228	0.7154	Delta Mass [ppm]

09/07/2023 08:32 AM	(7) Percolator:	-0.0110	0.2806	-0.1220	Absolute Delta Mass [Da]

09/07/2023 08:32 AM	(7) Percolator:	-1.0713	-1.0656	-0.5115	Absolute Delta Mass [ppm]

09/07/2023 08:32 AM	(7) Percolator:	0.4756	1.4784	0.8212	Peptide Length

09/07/2023 08:32 AM	(7) Percolator:	0.0000	0.0000	0.0000	Is z=1

09/07/2023 08:32 AM	(7) Percolator:	0.0081	0.3755	0.2231	Is z=2

09/07/2023 08:32 AM	(7) Percolator:	0.0354	-0.2600	-0.2105	Is z=3

09/07/2023 08:32 AM	(7) Percolator:	-0.1712	-0.3907	-0.1286	Is z=4

09/07/2023 08:32 AM	(7) Percolator:	0.1827	0.0983	0.1808	Is z=5

09/07/2023 08:32 AM	(7) Percolator:	0.0000	0.0000	0.0000	Is z>5

09/07/2023 08:32 AM	(7) Percolator:	-0.5368	-0.6076	0.0053	# Missed Cleavages

09/07/2023 08:32 AM	(7) Percolator:	0.0080	0.3126	0.1513	Log Peptides Matched

09/07/2023 08:32 AM	(7) Percolator:	-0.8036	-1.9664	-1.8099	Log Total Intensity

09/07/2023 08:32 AM	(7) Percolator:	-0.3280	-0.8283	-0.8903	Fraction Matched Intensity [%]

09/07/2023 08:32 AM	(7) Percolator:	-1.1434	-0.2778	-0.5101	Fragment Coverage Series A, B, C [%]

09/07/2023 08:32 AM	(7) Percolator:	0.1702	2.1784	1.2849	Fragment Coverage Series X, Y, Z [%]

09/07/2023 08:32 AM	(7) Percolator:	0.0542	0.1700	0.4899	Log Matched Fragment Series Intensities A, B, C

09/07/2023 08:32 AM	(7) Percolator:	0.3863	1.0514	0.5278	Log Matched Fragment Series Intensities X, Y, Z

09/07/2023 08:32 AM	(7) Percolator:	-0.7019	-0.2455	-0.0979	Longest Sequence Series A, B, C

09/07/2023 08:32 AM	(7) Percolator:	-0.4422	-1.0428	0.1006	Longest Sequence Series X, Y, Z

09/07/2023 08:32 AM	(7) Percolator:	-0.4399	-0.3908	-1.0205	IQR Fragment Delta Mass [Da]

09/07/2023 08:32 AM	(7) Percolator:	-0.5482	-0.0023	-0.3724	IQR Fragment Delta Mass [ppm]

09/07/2023 08:32 AM	(7) Percolator:	-1.4185	-0.1530	0.0362	Mean Fragment Delta Mass [Da]

09/07/2023 08:32 AM	(7) Percolator:	0.3502	0.1056	0.1415	Mean Fragment Delta Mass [ppm]

09/07/2023 08:32 AM	(7) Percolator:	-1.7261	-0.5839	0.0030	Mean Absolute Fragment Delta Mass [Da]

09/07/2023 08:32 AM	(7) Percolator:	1.0810	-0.3241	0.1676	Mean Absolute Fragment Delta Mass [ppm]

09/07/2023 08:32 AM	(7) Percolator:	-2.4250	-2.7613	-2.1724	m0

09/07/2023 08:32 AM	(7) Percolator:	Found 2570 test set PSMs with q<0.01.

09/07/2023 08:32 AM	(7) Percolator:	Selected best-scoring PSM per scan+expMass (target-decoy competition): 4961 target PSMs and 2027 decoy PSMs.

09/07/2023 08:32 AM	(7) Percolator:	Tossing out "redundant" PSMs keeping only the best scoring PSM for each unique peptide.

09/07/2023 08:32 AM	(7) Percolator:	Calculating q values.

09/07/2023 08:32 AM	(7) Percolator:	Final list yields 1205 target peptides with q<0.01.

09/07/2023 08:32 AM	(7) Percolator:	Calculating posterior error probabilities (PEPs).

09/07/2023 08:32 AM	(7) Percolator:	PSMId	score	q-value	posterior_error_prob	peptide	proteinIds

09/07/2023 08:32 AM	(7) Percolator:	Processing took 6.863 cpu seconds or 7 seconds wall clock time.

09/07/2023 08:32 AM	(7) Percolator:	Percolator (64Bit) process execution took 7.6 s.

09/07/2023 08:32 AM	(7) Percolator:	2628/28 high confident target/decoy peptides were found for Mascot (13).

09/07/2023 08:32 AM	(7) Percolator:	493/159 medium confident target/decoy peptides were found for Mascot (13).

09/07/2023 08:32 AM	(7) Percolator:	-- Total execution of Percolator (64Bit) for Mascot (13) took 37.4 s --

09/07/2023 08:32 AM	Job Execution:	----- Total Job execution took: 1 h 14 min. -----

09/07/2023 08:32 AM	Job Execution:	Finished D:\P1266_QE_FY2024\P1290\P1290-01_M4_WT-(2).msf

====================================================================================================

Validation

====================================================================================================

================================================================================

Consensus Step Validation

================================================================================

Result name: P1290-01_M4_WT

Result file: D:\P1266_QE_FY2024\P1290\P1290-01_M4_WT-(2).pdResult

Description: Result filtered for high confident peptides, with enhanced peptide and protein annotations. Add FASTA file with common contaminants to the Protein Marker node.

Workflow based on template: CWF_ProteinID_Comprehensive_Enhanced Annotation

Creation date: 9/7/2023 7:18:01 AM

Created with Discoverer version: 3.0.0.757

------------------------------------------------------------------

Peptide Validator nodes:

------------------------------------------------------------------

------------------------------------------------------------------

Processing node 2: Peptide Validator

------------------------------------------------------------------

1. General Validation Settings:

- Validation Mode: Automatic (Control peptide level error rate if possible)

- Target FDR (Strict) for PSMs: 0.01

- Target FDR (Relaxed) for PSMs: 0.05

- Target FDR (Strict) for Peptides: 0.01

- Target FDR (Relaxed) for Peptides: 0.05

2. Specific Validation Settings:

- Validation Based on: q-Value

- Target/Decoy Selection for PSM Level FDR Calculation Based on Score: Automatic

- Reset Confidences for Nodes without Decoy Search (Fixed score thresholds): False

Additional information:

--------------------------------

Used validation mode: 'Automatic (Control peptide level error rate if possible)'.

All PSMs have PEPs. Qvality will be used for peptides.

Updated peptide confidences using qvality.

------------------------------------------------------------------

Protein Validator nodes:

------------------------------------------------------------------

------------------------------------------------------------------

Processing node 7: Protein FDR Validator

------------------------------------------------------------------

1. Confidence Thresholds:

- Target FDR (Strict): 0.01

- Target FDR (Relaxed): 0.05

================================================================================

Processing Step A: Validation

================================================================================

Result name: P1290-01_M4_WT

Result file: D:\P1266_QE_FY2024\P1290\P1290-01_M4_WT-(2).msf

Description: Protein ID with Masco and Sequest HT Uniprott-Homo sapiens

Workflow based on template: ProteinID_Uniprot-Homo_sapiens

Creation date: 9/7/2023 7:17:58 AM

Created with Discoverer version: 3.0.0.757

------------------------------------------------------------------

Psm Validator nodes:

------------------------------------------------------------------

------------------------------------------------------------------

Processing node 6: Percolator

------------------------------------------------------------------

1. Target/Decoy Strategy:

- Target/Decoy Selection: Concatenated

- Validation based on: q-Value

2. Input Data:

- Maximum Delta Cn: 0.05

- Maximum Rank: 0

3. FDR Targets:

- Target FDR (Strict): 0.01

- Target FDR (Relaxed): 0.05

------------------------------------------------------------------

Validation for Processing Node: Sequest HT (12)

------------------------------------------------------------------

Percolator Output:

--------------------------------

Results for Sequest HT (12):

Iteration 1:	Estimated 2677 PSMs with q<0.01

Iteration 2:	Estimated 2814 PSMs with q<0.01

Iteration 3:	Estimated 2899 PSMs with q<0.01

Iteration 4:	Estimated 2944 PSMs with q<0.01

Iteration 5:	Estimated 2965 PSMs with q<0.01

Iteration 6:	Estimated 2984 PSMs with q<0.01

Iteration 7:	Estimated 3008 PSMs with q<0.01

Iteration 8:	Estimated 3018 PSMs with q<0.01

Iteration 9:	Estimated 3013 PSMs with q<0.01

Iteration 10:	Estimated 3029 PSMs with q<0.01

Learned normalized SVM weights for the 3 cross-validation splits:

Split1	Split2	Split3	FeatureName

-0.1214	-0.5845	0.3962	XCorr

1.1966	0.9872	0.1197	Delta Cn From Second PSM

4.7708	5.2485	7.4426	Binomial Score

-0.1218	0.0757	0.6391	Isolation Interference [%]

-0.3237	-0.4200	0.7351	MH+ [Da]

0.6596	0.3378	-0.9670	Delta Mass [Da]

-0.1097	-0.1460	0.6200	Delta Mass [ppm]

-1.3003	-0.5487	-0.9278	Absolute Delta Mass [Da]

0.3047	-0.2572	0.0000	Absolute Delta Mass [ppm]

-1.2616	-0.2188	-1.6587	Peptide Length

-0.4192	-0.6960	-0.4332	Is z=1

-0.1258	0.1870	0.3300	Is z=2

0.2982	0.3246	-0.1399	Is z=3

0.6619	0.3247	0.1712	Is z=4

0.2024	0.0727	0.3219	Is z=5

0.1179	0.0605	0.1331	Is z>5

-0.5739	-0.7231	-0.8954	# Missed Cleavages

0.0251	-0.2496	-0.2845	Log Peptides Matched

-1.4882	-1.2749	-3.1346	Log Total Intensity

-0.2360	-0.3618	-0.5276	Fraction Matched Intensity [%]

-1.5805	-0.9701	-1.5945	Fragment Coverage Series A, B, C [%]

-0.4792	-0.7276	-0.8875	Fragment Coverage Series X, Y, Z [%]

0.0420	-0.1278	0.3492	Log Matched Fragment Series Intensities A, B, C

-0.8321	-0.7546	0.3650	Log Matched Fragment Series Intensities X, Y, Z

0.0750	-0.4848	-0.9895	Longest Sequence Series A, B, C

-0.0469	0.0777	0.2303	Longest Sequence Series X, Y, Z

-0.1753	-0.2543	-0.3809	IQR Fragment Delta Mass [Da]

-1.3773	0.4361	-1.7740	IQR Fragment Delta Mass [ppm]

0.0476	-0.4150	-1.4485	Mean Fragment Delta Mass [Da]

0.1759	0.4084	1.7877	Mean Fragment Delta Mass [ppm]

-0.6523	-2.5277	-0.1861	Mean Absolute Fragment Delta Mass [Da]

0.4154	1.1357	-1.4492	Mean Absolute Fragment Delta Mass [ppm]

-5.8933	-6.2708	-9.6580	m0

------------------------------------------------------------------

Processing node 7: Percolator

------------------------------------------------------------------

1. Target/Decoy Strategy:

- Target/Decoy Selection: Concatenated

- Validation based on: q-Value

2. Input Data:

- Maximum Delta Cn: 0.05

- Maximum Rank: 0

3. FDR Targets:

- Target FDR (Strict): 0.01

- Target FDR (Relaxed): 0.05

------------------------------------------------------------------

Validation for Processing Node: Mascot (13)

------------------------------------------------------------------

Percolator Output:

--------------------------------

Results for Mascot (13):

Iteration 1:	Estimated 2609 PSMs with q<0.01

Iteration 2:	Estimated 2708 PSMs with q<0.01

Iteration 3:	Estimated 2743 PSMs with q<0.01

Iteration 4:	Estimated 2752 PSMs with q<0.01

Iteration 5:	Estimated 2771 PSMs with q<0.01

Iteration 6:	Estimated 2781 PSMs with q<0.01

Iteration 7:	Estimated 2780 PSMs with q<0.01

Iteration 8:	Estimated 2782 PSMs with q<0.01

Iteration 9:	Estimated 2781 PSMs with q<0.01

Iteration 10:	Estimated 2780 PSMs with q<0.01

Learned normalized SVM weights for the 3 cross-validation splits:

Split1	Split2	Split3	FeatureName

0.6287	0.7846	0.2906	Delta Cn From Second PSM

3.5550	1.9542	2.3262	Binomial Score

0.3168	0.2527	0.2663	Isolation Interference [%]

-0.0693	-0.2743	-0.3336	MH+ [Da]

0.2863	-0.2454	-0.6811	Delta Mass [Da]

-0.2079	0.1228	0.7154	Delta Mass [ppm]

-0.0110	0.2806	-0.1220	Absolute Delta Mass [Da]

-1.0713	-1.0656	-0.5115	Absolute Delta Mass [ppm]

0.4756	1.4784	0.8212	Peptide Length

0.0000	0.0000	0.0000	Is z=1

0.0081	0.3755	0.2231	Is z=2

0.0354	-0.2600	-0.2105	Is z=3

-0.1712	-0.3907	-0.1286	Is z=4

0.1827	0.0983	0.1808	Is z=5

0.0000	0.0000	0.0000	Is z>5

-0.5368	-0.6076	0.0053	# Missed Cleavages

0.0080	0.3126	0.1513	Log Peptides Matched

-0.8036	-1.9664	-1.8099	Log Total Intensity

-0.3280	-0.8283	-0.8903	Fraction Matched Intensity [%]

-1.1434	-0.2778	-0.5101	Fragment Coverage Series A, B, C [%]

0.1702	2.1784	1.2849	Fragment Coverage Series X, Y, Z [%]

0.0542	0.1700	0.4899	Log Matched Fragment Series Intensities A, B, C

0.3863	1.0514	0.5278	Log Matched Fragment Series Intensities X, Y, Z

-0.7019	-0.2455	-0.0979	Longest Sequence Series A, B, C

-0.4422	-1.0428	0.1006	Longest Sequence Series X, Y, Z

-0.4399	-0.3908	-1.0205	IQR Fragment Delta Mass [Da]

-0.5482	-0.0023	-0.3724	IQR Fragment Delta Mass [ppm]

-1.4185	-0.1530	0.0362	Mean Fragment Delta Mass [Da]

0.3502	0.1056	0.1415	Mean Fragment Delta Mass [ppm]

-1.7261	-0.5839	0.0030	Mean Absolute Fragment Delta Mass [Da]

1.0810	-0.3241	0.1676	Mean Absolute Fragment Delta Mass [ppm]

-2.4250	-2.7613	-2.1724	m0

================================================================================

Filters and Counts

================================================================================

------------------------------------------------------------------

Applied display filters:

------------------------------------------------------------------

This file contains the following filters:

Row Filter for Annotation Protein Groups:

------------------------------------

GroupAnnotationLevel is equal to Leaf

------------------------------------

Row Filter for Pathway Protein Groups:

------------------------------------

GroupPathwayLevel is equal to Leaf

------------------------------------

Row Filter for Proteins:

------------------------------------

Master is equal to Master

------------------------------------

Row Filter for Input Files:

------------------------------------

FileName does not end with msf

------------------------------------

------------------------------------------------------------------

Number of result items:

------------------------------------------------------------------

Proteins:

331 filtered / 3255 included / 38228 total

Protein Groups:

331 included / 335 total

Peptide Groups:

1314 included / 9762 total

PSMs:

5186 included / 20142 total

MS/MS Spectrum Info:

18202 total

Input Files:

1 filtered / 2 included / 2 total

Study Information:

1 total

Specialized Traces:

2 total

Annotation Protein Groups:

1189 filtered / 5411 included / 5411 total

Pathway Protein Groups:

726 filtered / 824 included / 824 total

Result Statistics:

202 total

================================================================================

FDR Values for Entire Result

================================================================================

------------------------------------------------------------------

Actual estimated FDR values (without applied display filters):

------------------------------------------------------------------

These estimated FDR values are based on simple counting of target and decoy items

and may divert from the target FDR values you have set in the validation nodes

used during workflow processing.

Usually the values will be slightly more conservative than using validation

based on linear discriminant analysis or other sophisticated methods.

--------------------------------

High confident results:

--------------------------------

0.003 (5186 targets, 15 decoys) for Peptide-Spectrum Matches

0.008 (1314 targets, 11 decoys) for Peptide Groups

0.007 (3255 targets, 24 decoys) for Proteins

(No confidence was assigned to Protein Groups)

--------------------------------

Medium and High confident results:

--------------------------------

0.003 (5186 targets, 15 decoys) for Peptide-Spectrum Matches

0.008 (1314 targets, 11 decoys) for Peptide Groups

0.007 (3255 targets, 24 decoys) for Proteins

(No confidence was assigned to Protein Groups)

--------------------------------

Whole dataset:

--------------------------------

0.003 (5186 targets, 15 decoys) for Peptide-Spectrum Matches

0.008 (1314 targets, 11 decoys) for Peptide Groups

0.007 (3255 targets, 24 decoys) for Proteins

0.033 (331 targets, 11 decoys) for Protein Groups

====================================================================================================

Configuration

====================================================================================================

================================================================================

Consensus Workflow Configuration

================================================================================

Result name: P1290-01_M4_WT

Result file: D:\P1266_QE_FY2024\P1290\P1290-01_M4_WT-(2).pdResult

Description: Result filtered for high confident peptides, with enhanced peptide and protein annotations. Add FASTA file with common contaminants to the Protein Marker node.

Workflow based on template: CWF_ProteinID_Comprehensive_Enhanced Annotation

Creation date: 9/7/2023 7:18:01 AM

Created with Discoverer version: 3.0.0.757

------------------------------------------------------------------

Configuration for: MSF Files

------------------------------------------------------------------

Scores:

- PSM scores (Hidden):

Mascot: Ions Score

Sequest HT: XCorr

SEQUEST: XCorr

MSPepSearch: dot Score

MSPepSearch: rev-dot Score

MSPepSearch: MSPepSearch Score

PMI-Byonic: |Log Prob|

PMI-Byonic: Byonic Score

MS Amanda: Amanda Score

------------------------------------------------------------------

Configuration for: Protein Scorer

------------------------------------------------------------------

Configuration Settings for Protein Score 'SequestSummationScore':

Protein Scoring Options:

- Peptide Relevance Factor: 0.4

Configuration Settings for Protein Score 'MascotSummationScore':

Protein Scoring Options:

- Use MudPIT Scoring: Automatic

Mascot protein score is calculated as MudPIT score

------------------------------------------------------------------

Configuration for: Protein Annotation

------------------------------------------------------------------

1. Annotation Server:

- Annotation Service URL: https://proteinannotationservice.thermofisher.com/

- Number of attempts to submit the annotation request: 3

- Time interval between attempts to submit the annotation request [sec]: 90

- Timeout of the annotation request [min]: 15

2. Annotation/Pathway Groups:

- Max. Database Size: 500000

------------------------------------------------------------------

Configuration for: Display Settings

------------------------------------------------------------------

Default Display Filter:

- Default Filter Set (Hidden):

\###	Master Proteins Default Filter:

\###	Row Filter for TargetProtein:

\###	Master is equal to Master

\###

'magellan filter set' 1 'MasterFilter.filterset' Filter 'TargetProtein' FilterProperties 1 'FilterConditionProperties/FilterScope' 'FilterConditionProperties/FilterScopeValueMainGrid' 1 NARY_AND 1 = FilterConditionProperties 1 'NamedComparableFilterCondition/DisplayPropertyHint' 'Master' property 'Thermo.PD.EntityDataFramework.MasterProteinAssessment, Thermo.Magellan.EntityDataFramework' 'IsMasterProtein' constant 'Thermo.PD.EntityDataFramework.MasterProteinAssessment, Thermo.Magellan.EntityDataFramework' 'IsMasterProtein'

Default Layout:

- Default Layout: (not specified)

================================================================================

Processing Workflow A: Configuration

================================================================================

Result name: P1290-01_M4_WT

Result file: D:\P1266_QE_FY2024\P1290\P1290-01_M4_WT-(2).msf

Description: Protein ID with Masco and Sequest HT Uniprott-Homo sapiens

Workflow based on template: ProteinID_Uniprot-Homo_sapiens

Creation date: 9/7/2023 7:17:58 AM

Created with Discoverer version: 3.0.0.757

------------------------------------------------------------------

Configuration for: Spectrum Files RC

------------------------------------------------------------------

Search settings:

- Ion Series settings (Hidden):

CID: by

HCD: by

ECD: cyz

ETD: cyz

EThcD: bcyz

UVPD: abxyz

------------------------------------------------------------------

Configuration for: Sequest HT

------------------------------------------------------------------

1. Workload Level:

- Automatic: True

- Number of Spectra Processed At Once: 3000

- Number of Parallel Tasks: 0

2. XCorr Confidence Thresholds (low-resolution data):

- z=1: High Confidence XCorr: 1.5

- z=1: Medium Confidence XCorr: 0.7

- z=2: High Confidence XCorr: 2

- z=2: Medium Confidence XCorr: 0.9

- z=3: High Confidence XCorr: 2.5

- z=3: Medium Confidence XCorr: 1.2

- z>=4: High Confidence XCorr: 3

- z>=4: Medium Confidence XCorr: 1.5

3. XCorr Confidence Thresholds (high-resolution data):

- z=1: High Confidence XCorr: 1.2

- z=1: Medium Confidence XCorr: 0.7

- z=2: High Confidence XCorr: 1.9

- z=2: Medium Confidence XCorr: 0.8

- z=3: High Confidence XCorr: 2.3

- z=3: Medium Confidence XCorr: 1

- z>=4: High Confidence XCorr: 2.6

- z>=4: Medium Confidence XCorr: 1.2

------------------------------------------------------------------

Configuration for: Mascot

------------------------------------------------------------------

1. Default Confidence Thresholds:

- Significance High: 0.01

- Significance Middle: 0.05

1. Mascot Server:

- Max. MGF File Size [MB]: 500

- Time interval between attempts to submit a search [sec]: 90

- Number of attempts: 20

- Mascot Server URL: http://localhost/mascot/

2. Mascot Server Authentication:

- Mascot User Name: (not specified)

- Mascot Server Password: (not specified)

3. Web Server Authentication:

- User Name: (not specified)

- Password: (not specified)
